# Highly Polymorphic Microsatellite Markers for the Assessment of Male Reproductive Skew and Genetic Variation in Critically Endangered Crested Macaques (*Macaca nigra*)

**DOI:** 10.1007/s10764-017-9973-x

**Published:** 2017-07-21

**Authors:** Antje Engelhardt, Laura Muniz, Dyah Perwitasari-Farajallah, Anja Widdig

**Affiliations:** 1 School of Natural Sciences and Psychology, Liverpool John Moores University, L3 3AF, Liverpool, UK; 2 Junior Research Group of Primate Sexual Selection, German Primate Center, 37077 Göttingen, Germany; 3 Courant Research Center Evolution of Social Behavior, Georg August University, 37077 Göttingen, Germany; 4 Junior Research Group of Primate Kin Selection, Department of Primatology, Max-Planck Institute for Evolutionary Anthropology, D-04103 Leipzig, Germany; 5 Research Group of Behavioural Ecology, Institute of Biology, University of Leipzig, 04103 Leipzig, Germany; 6 Primate Research Centre, Bogor Agricultural University, Bogor, Indonesia; 7 Department of Biology, Faculty of Mathematics and Natural Sciences, Bogor Agricultural University, Bogor, Indonesia; 8 German Center for Integrative Biodiversity Research, 04103 Leipzig, Germany

**Keywords:** Conservation, Genetic variation, Heterozygosity, Inbreeding, *Macaca nigra*, Microsatellite markers, Paternity, Reproductive skew, Sulawesi

## Abstract

Genetic analyses based on noninvasively collected samples have become an important tool for evolutionary biology and conservation. Crested macaques (*Macaca nigra*), endemic to Sulawesi, Indonesia, are important for our understanding of primate evolution as Sulawesi macaques represent an exceptional example of primate adaptive radiation. Crested macaques are also Critically Endangered. However, to date we know very little about their genetics. The aim of our study was to find and validate microsatellite markers useful for evolutionary, conservation, and other genetic studies on wild crested macaques. Using fecal samples of 176 wild macaques living in the Tangkoko Reserve, Sulawesi, we identified 12 polymorphic microsatellite loci through cross-species polymerase chain reaction amplification with later modification of some of these primers. We tested their suitability by investigating and exploring patterns of paternity, observed heterozygosity, and evidence for inbreeding. We assigned paternity to 63 of 65 infants with high confidence. Among cases with solved paternity, we found no evidence of extragroup paternity and natal breeding. We found a relatively steep male reproductive skew *B* index of 0.330 ± 0.267; mean ± SD) and mean alpha paternity of 65% per year with large variation across groups and years (29–100%). Finally, we detected an excess in observed heterozygosity and no evidence of inbreeding across our three study groups, with an observed heterozygosity of 0.766 ± 0.059 and expected heterozygosity of 0.708 ± 0.059, and an inbreeding coefficient of −0.082 ± 0.035. Our results indicate that the selected markers are useful for genetic studies on wild crested macaques, and possibly also on other Sulawesi and closely related macaques. They further suggest that the Tangkoko population of crested macaques is still genetically variable despite its small size, isolation, and the species’ reproductive patterns. This gives us hope that other endangered primate species living in small, isolated populations may also retain a healthy gene pool, at least in the short term.

**Electronic supplementary material:**

The online version of this article (doi:10.1007/s10764-017-9973-x) contains supplementary material, which is available to authorized users.

## Introduction

The development of genetic analyses has revolutionized various fields in the medical and life sciences. More recently, genetic analyses based on naturally dropped animal waste such as fur, feathers, and feces have created new opportunities for studies of wildlife under natural conditions, particularly endangered and/or elusive species, and other species in which capturing constitutes an ethical problem (Waits and Paetkau 2005). Potential applications of genetic analyses for field studies include examining the occurrence, distribution, and history of species (Hewitt 2000; Leonard 2008; Ram *et al.* 2015); investigating taxonomic relationships and speciation (Tosi et al. 2003); assessing hybridization (Charpentier *et al.* 2012; Godinho *et al.* 2015; Roos *et al.* 2011); determining the level of heterozygosity, gene flow, and the risk of inbreeding depression of isolated populations (Knief *et al.* 2015; Luikart *et al.* 1998; Nürnberg *et al.* 1998; Ram *et al.* 2015; Widdig *et al.* 2004, 2017); monitoring population developments and movements (Nowak *et al.* 2014); identifying species (Harms *et al.* 2015); and studying reproductive patterns (Engelhardt *et al.* 2006; Syrůčková *et al.* 2015; Widdig *et al.* 2004) and kin relationships in groups and populations (Montague *et al.* 2014; van Horn *et al.* 2008). Hence, studies of evolutionary biology, biogeography, and behavioral ecology greatly benefit from the availability of genetic analyses based on noninvasively collected samples, as does conservation management (Schwartz *et al.* 2007). The genetic markers used in such studies often need to be specified for the species in question, although the same markers can be used for closely related species.

Genetic markers are not yet available but would be very important for the Sulawesi macaques. The seven species of macaques on the island of Sulawesi (*Macaca brunnescens*, *M. hecki*, *M. maurus*, *M. nigra*, *M. nigrescens*, *M. ochreata*, *M. tonkeana*), the main island of the Wallacea biodiversity hotspot, are an important group for our understanding of primate evolution. Endemic to the island, they are a prominent example of primate adaptive radiation and speciation in relation to the processes of geological change and colonization of new areas (Groves 1980). All seven species live in different habitats with only narrow overlapping contact zones, in which interbreeding occurs (Evans *et al.* 2003; Fooden 1982). Furthermore, Sulawesi macaques are the only macaques classified as extremely socially tolerant, with high conciliatory tendencies and low degrees of power asymmetries (Thierry 2004; Thierry *et al.* 2000). Few studies have investigated Sulawesi macaques in the wild because their habitat is very difficult to access. However, the rainforests of Sulawesi are now more accessible, and the infrastructure on Sulawesi has improved, facilitating studies of Sulawesi wildlife. However, with these developments, the natural habitat of the macaques is shrinking and fragmented, and heavily exploited by humans. As a result, all seven Sulawesi macaques are in danger of extinction to various degrees (IUCN 2016). Given the precarious situation and geographic isolation of Sulawesi macaques, genetic studies on these species are important not only for our understanding of primate evolution (Evans *et al.* 1999, 2003), but also for their conservation management.

Crested macaques, *Macaca nigra*, are found only on the northern tip of Sulawesi. Habitat degradation and bushmeat hunting have brought this species to the edge of extinction, with the largest remaining population of fewer than 2000 individuals seemingly occurring in Tangkoko Reserve (Melfi 2010; Palacios *et al.* 2012). There are at least two reasons why we need genetic studies of crested macaques. First, crested macaques are of particular interest for better understanding primate evolution because the species possesses features not found in any of the other Sulawesi macaques. For example, other Sulawesi macaques live in groups of ≤40 individuals, while crested macaques live in large groups sometimes containing >100 individuals (Marty *et al.* 2015; Riley 2010). Despite the large group size, crested macaques seem to be an extreme case in terms of male–male reproductive competition, with males fighting fiercely for dominance (Marty 2015) and dominant males able to monopolize matings with fertile females (Engelhardt *et al. unpubl. Data*). The male hierarchy, particularly the first three ranks, is so important that it is clearly signaled in the occurrence and structure of loud calls (Neumann *et al.* 2010). Based on these observations, we can expect male reproductive skew in favor of dominant males, as observed in other primates (Widdig 2013), meaning that many infants sired during a male’s tenure will share paternal genes. At the same time, the male hierarchy in crested macaques is highly dynamic (Neumann *et al.* 2011), with high takeover rates resulting in a mean alpha tenure of only 12 mo (Marty *et al.* 2015), so infants born in different years often have different fathers. However, the genetic consequences of male reproductive strategies at the population level remain unclear, as no study has investigated male reproduction in crested macaques using genetic data. High reproductive skew may result in lower genetic variation, as only few, top-ranking males pass on their genes to the next generation; however, the high takeover rate in alpha male position may counteract the effect of reproductive monopolization and contribute to the maintenance of genetic variation in the population.

The second reason why we need genetic studies of crested macaques is that they are the most threatened Sulawesi macaques, and are Critically Endangered (IUCN 2016). Genetic studies of crested macaques are limited to mitochondrial and autosomal DNA phylogeny (Evans *et al.* 1999, 2003). The degree of gene flow and the risk of inbreeding depression remain unclear for the remaining populations of crested macaques. Furthermore, many animals, rescued from illegal captivity and currently held in sanctuaries, await release into the wild. We cannot determine the genetic value of these individuals for wild populations until genetic evaluations are feasible. It is important to detect hybrids among these rescued individuals to avoid releasing them into hybrid-free populations. Finally, we need to understand the genetic variation in the largest population remaining in its natural distribution range, Tangkoko. This information is highly relevant to conservation management. However, we still lack genetic markers useful for such analyses in crested macaques.

The first aim of this study was to identify highly polymorphic microsatellite (short tandem repeats [STR]) markers for reliable genotyping in crested macaques. Testing primers originally designed for other, usually closely related species (cross-species amplification) is often the cheapest and fastest way to define a set of useful markers. Our second aim was to test the suitability of the selected markers. To do this, we determined maker polymorphism and checked for Hardy–Weinberg equilibrium and Mendelian inheritance between known mother–offspring pairs. Our third aim was to assign paternity to the Tangkoko animals and determine the degree of male reproductive skew (using the *B* index; Nonacs 2000, 2003), which we predicted to be high based on the observed mating skew (Engelhardt *et al. unpubl. Data*). We predicted a low degree of extragroup paternities and natal breeding, given that a few males monopolize all receptive females. As a final aim, we investigated whether this isolated population shows signs of loss of heterozygosity by comparing observed and expected heterozygosity, as well as evaluated estimates of inbreeding in this fragmented population.

## Methods

### Study Population

We studied crested macaques at Tangkoko Reserve (1 N 32′39′′, 125E 12′42′′), North Sulawesi, Indonesia. A recent study in the reserve estimated the population size to be fewer than 2000 individuals (Palacios *et al.* 2012). Tangkoko Reserve borders another nature reserve, Duasudara Reserve, but is disconnected from all other forested areas in North Sulawesi. The number of crested macaques currently living in Duasudara Reserve is unknown, but preliminary data suggest it to be very low (Palacios *et al.* 2012). However, there may be some genetic exchange between individuals in the two reserves.

As in other macaque species, female crested macaques stay in their natal groups for life, forming matrilines, while males emigrate from their natal group. Males are fully grown when they emigrate and frequently challenge alpha males in another group when immigrating (Marty *et al.* 2015). Although females give birth year-round, they are moderately seasonal (Marty *et al.* 2016), with an interbirth interval of ca. 22 mo (Marty *et al.* 2015).

The *Macaca nigra* Project observes three groups (R1, R2, PB) almost daily (R1 and R2 since 2006 and PB since 2008 until the present) collecting behavioral data including aggressive interactions and their outcomes through focal animal and ad libitum sampling (Altmann 1974). We also recorded births, deaths, and migration events. All adult individuals and sampled infants were individually recognized. During our study period, the home range of group R1 overlapped with that of R2 and PB. All three groups also overlapped with other, nonstudy groups. We individually recognized all adult individuals of the three groups as well as infants used for paternity analysis in this study. Group size ranged between 36 and >100 individuals across years.

We used the David score (de Vries *et al.* 2006) to assess dominance rank on a matrix of proportions of wins calculated for each male–male dyad. We calculated David scores using the package Steepness (Leiva and de Vries 2011) in R (R Team 2009). We used either hormonal data or data of sex skin swelling size to assess conception windows (for details see Higham *et al.* 2012). In addition, we combined demographic and hierarchy data to compute annual alpha tenure.

### Sample Collection

We collected noninvasive fecal samples immediately after defecation from 176 individually recognized individuals from all three groups from 2006 onwards. We collected up to three samples for each individual. Following the two-step alcohol–silica storage protocol (Nsubuga *et al.* 2004), we placed 1–2 g from the surface of fresh feces into a 50-mL plastic tube filled with 30 mL of 99% ethanol for ≥24 h. Subsequently, we placed the sample in another tube filled with 30 mL of silica beads and stored it at room temperature until extraction. In a few cases, we collected ejaculates from males, which we stored in 98% ethanol at room temperature until extraction. We considered any adult males present or immigrating into our study groups during our study period as potential sires. We defined adult males as larger than fully grown females, with fully erupted canines and completely descended testes. We obtained DNA samples for 54 of 56 potential sires (96%), including all adult males present in one of the three study groups since 2006. For one male, however, we only obtained one sample and the DNA obtained was of such low quality that it amplified successfully at only nine loci.

We also obtained fecal and blood samples during regular health checks of seven crested macaques (one of each per individual) from Dublin Zoo.

### DNA Extraction

We extracted DNA from 100 to 150 mg of feces with the GEN-IAL® all-tissue DNA extraction kit following the manufacturer’s instructions with the exception that we eluted DNA in distilled water.

### Identification of Polymorphic Markers

*Testing Potential Markers* via *Cross-Species Amplification* We tested 39 microsatellite loci previously described to be polymorphic in rhesus (*Macaca mulatta*), long-tailed (*M. fascicularis*), and Barbary (*M. sylvanus*) macaques (Brauch *et al.* 2008; Engelhardt *et al.* 2006; Nürnberg *et al.* 1998; Widdig *et al.* 2017) for allele amplification and polymorphism with a set of nine different polymerase chain reaction (PCR) conditions to increase the chances of successful cross-species amplification (cf. Moore *et al.* 1991) in crested macaques. For this, we combined three different magnesium salt concentrations (1.5 mM, 2.0 mM, 2.5 mM) with three different annealing temperatures (56, 58, and 60 °C or 51, 53, and 55 °C, depending on primer pair). In this step, we used a high-quality pooled DNA sample (from blood) from the seven Dublin Zoo individuals. When we obtained a readable product for a primer pair, we selected the condition that yielded the highest concentration of the specific product and fewer stutters for individual genotyping and polymorphism check. We included the matching fecal and blood samples from the seven Dublin crested macaques to confirm that genotypes obtained from fecal samples matched those from blood samples. Finally, we tested Mendelian inheritance by individually amplifying DNAs from known mother–offspring pairs.

#### Genotyping and Determination of Alleles

To genotype the 176 subjects, we used a two-step multiplex PCR approach (modified from Arandjelovic *et al.* 2009). First, we amplified all loci in a multiplex approach using 4 μL of DNA extract (diluted 1:50 or 1:100), 0.2 μL of H_2_O, 2 μL of 10× Master *Taq* Buffer with Mg^2+^ (5 PRIME®, 500 mM KCl, 100 mM Tris-HCl pH 8.3, 15 mM Mg(OAc)2), 2 μL of 5 *Taq*Master PCR Enhancer (5 PRIME®), 0.8 μL of dNTPs (10 mM), 1.2 μL of MgCl_2_ (25 mM), 0.4 μL (10 pmol) of 12 unlabeled forward and reverse primers, respectively, and 0.2 μL of 5 PRIME® *Taq* DNA polymerase (5 U/μL, enzyme storage buffer: 20 mM Tris·HCl pH 8.0, 100 mM KCl, 0.1 mM EDTA, 1 mM dithiothreitol (DTT), 50% glycerol, 0.5% Tween®20, 0.5% Igepal®CA-630) in an Eppendorf® Master Cycler Gradient. We started with 2 min of denaturation at 94 °C, then ran 30 cycles of 20 s denaturation at 94 °C, 30 s of annealing at 54 °C, 30 s of elongation at 70 °C, and ended with 10 min of final elongation at 70 °C. Following the multiplex approach, we ran singleplex PCRs to amplify one locus at a time using a similar protocol with specific annealing temperatures per primer pair (Table I). Specifically, we amplified 1 μL of multiplex PCR with 13.7 μL of H_2_O, 2 μL of 10× Master *Taq* Buffer with Mg^2+^ (5 PRIME®, 500 mM KCl, 100 mM 206 Tris-HCl pH 8.3, 15 mM Mg(OAc)2), 0.5 μL of 5× *Taq*Master PCR Enhancer (5 PRIME®), 0.8 μL of dNTPs (10 mM), 0.8 μL of MgCl_2_ (25 mM), 0.5 μL (10 pmol) of each primer labeled (HEX or FAM) forward and unlabeled reverse, and 0.2 μL of 5 PRIME® *Taq* DNA Polymerase (5 U/μL, enzyme storage buffer: 20 mM Tris·HCl pH 8.0, 100 mM KCl, 0.1 mM EDTA, 1 mM DTT, 50% glycerol, 0.5% Tween®20, 0.5% Igepal®CA-630). We prepared singleplex PCR products for analysis by diluting PCR products between 1:25 and 1:500, and mixing 1.5 μL of diluted product into 14 μL of Hi-Di formamide buffer mixed with a size standard (HD400 from Applied Biosystems®). Finally, we ran amplicons on an ABI 3130xL sequencer and determined allele sizes with PeakScanner (Applied Biosystems®).

**Table I:** Characterization of 12 primer pairs for amplifying polymorphic microsatellite loci in crested macaques with PCR conditions, deviation from Hardy–Weinberg equilibrium, and estimated null allele frequency

Locus	Repeat pattern	Length of PCR product Dublin Zoo (bp)	Length of PCR product Tangkoko (bp)	Annealing temperature (°C)	Hardy–Weinberg deviation	Estimated null allele frequency	Primer sequence (5′–3′) (including modified primers)	Reference
D1S548	Tetra	181–201	185–209	58	n.s.	−0.0394	F: GAACTCATTGGCAAAAGGAA R: GCCTCTTTGTTGCAGTGATT	Lathuillière *et al.* 2001
D3S1768	Tetra	129–137	129–157	58	n.s.	−0.046	F: GGTTGCTGCCAAAGATTAGA R: AACTACATGATTCTAGCACA	Lathuillière *et al.* 2001
D5S1457	Tetra	123, 127, 131	123–139	60	n.s.	−0.0609	F: TAGGTTCTGGGCATGTCTG R: TTGCTTGGCACACTTCAGG	Bayes *et al.* 2000
D6S493*	Tetra	261–269^b^	139–159^c^	58	n.s.	−0.0374	F: GCAACAGTTTATGCTAAAGC R: TTCCATGGCAGAAATTGTTT	Nürnberg *et al.* 1998
D6S501*	Tetra	163–179^b^	129–145^c^	58	n.s.	−0.0345	F: GCTGGAAACTGATAAGGGCT R: CTTTATCTTTAATATAGGATTATTGG	Lathuillière *et al.* 2001
D7S2204	Tetra	171–247	220–268	58	n.s.	−0.0579	F: TCATGACAAAACAGAAATTAAGTG R: AGTAAATGGAATTGCTTGTTACC	Lathuillière *et al.* 2001
D10S1432	Tetra	137–145	132–148	58	n.s.	−0.0773	F: CAGTGGACACTAAACACAATCC R: TAGATTATCTAAATGGTGGATTTCC	Lathuillière *et al.* 2001
D11S925	Di	205–221	179–237	60	n.s.	−0.0379	F: GAACCAAGGTCGTAAGTCC R: TAGACCATTATGGGGGCAAA	Lathuillière *et al.* 2001
D12S67^a^	Tetra	135,177–193^b^	159–185^c^	58	n.s.	−0.0262	F: GCAACAGTTTATGCTAAAGC R: TGTTGTTCAAGGGTCAAATG	Nürnberg *et al.* 1998
D13S765^a^	Tetra	220, 224, 232^b^	137–165^c^	58	n.s.	−0.0512	F: TGTAACTTACTTCAAATGGCTCA R: ATTTACCTAACATTTCACCCATC	Zhang *et al.* 2001
D14S255^a^	Di	173–185^b^	91–113^c^	60	n.s.	−0.0142	F: AGCTTCCAATACCTCACCAA R: CTCTTAGTGGTCATTCTCAC	Nürnberg *et al.* 1998
D18S536	Tetra	144–152	144–164	58	n.s.	−0.0491	F: ATTATCACTGGTGTTAGTCCT R: CACAGTTGTGTGAGCCAGT	Kümmerli and Martin 2005

F indicates forward primers and R indicates reverse primers. n.s. = no significant deviation.

^a^Primers of this marker were modified to be specific to crested macaques.

^b^Before primer modification.

^c^After primer modification.

We analyzed the samples in two laboratories (German Primate Center and Max-Planck Institute for Evolutionary Anthropology), with the identical protocols and equipment. We compared 5 individuals genotyped in both laboratories on the 12 markers and found genotype inconsistency in 2 of the 118 alleles, giving an error rate of 0.016.

#### Modification of Markers

Many of the tested primer pairs produced unspecific products, typically detected as three or more differently sized amplicons resulting from the simultaneous amplification of two or more loci (Smith *et al.* 2000). Since only seven markers repeatedly produced up to two alleles per individual, we modified specific primers for crested macaques for the other five identified markers (Table I). For this, we located sequences closer to the repetitive sequence than the respective original primers. We then generated ligation of PCR products of the specific microsatellites into plasmid vector pCR®2.1-TOPO® with the TOPO TA Cloning®Kit (Invitrogen, Carlsbad, CA, USA) followed by colony hybridization as described in Takenaka *et al.* (1993). We isolated plasmids containing the specific repeats from *E. coli* using the QIAprep Spin Miniprep Kit (Qiagen). Next, we conducted fluorescent sequencing with the Autocycle Sequence Kit Big Dye in the ABI Prism 3100 sequencer (Applied Biosystems, Foster City, CA, USA). Finally, we synthesized the selected primer sequences with Thermo Hybaid, Ulm, Germany (Table I). There may be further additional suitable markers among those we tested, particularly if they are optimized for the species.

#### Final Marker Selection

We selected the 12 best markers using the following criteria: 1) we preferred markers with tetra-repeats over di-repeats, 2) amplification success at least 50%, 3) markers that were polymorphic with at least three alleles), and 4) markers with reliable allele size scoring (no or few stutters/multiple peaks). As fecal samples contain only a small amount of DNA and a high level of allelic dropouts (Bayes *et al.* 2000), we genotyped three independent fecal samples for each individual if available. Based on previous studies (Brauch *et al.* 2008; Engelhardt *et al.* 2006), we accepted a heterozygous genotype only if two different samples of the same individual showed the same result in at least four amplifications; likewise, we accepted a homozygous genotype if it was consistent in at least six amplifications (Taberlet *et al.* 1996). If we identified a third allele during analysis, we doubled the number of amplifications.

### Testing the Suitability of Selected Markers

#### Polymorphic Information Content and Hardy–Weinberg Equilibrium

To investigate the suitability of our markers, we first calculated the polymorphic information content (PIC), an estimate of the discriminating power of markers (ranging from 0 to 1, from no allelic variation to only new alleles) (Botstein *et al.* 1980). We also tested markers for deviation from Hardy–Weinberg equilibrium (HWE). We considered that deviation from the HWE would indicate genotyping problems, such as segregating null alleles or incorrectly distinguished alleles.

#### Assessment of Mendelian Inheritance

We investigated whether behavioral mothers (known from behavioural observations, i.e. association and nursing) were also the genetic mothers by testing Mendelian inheritance for 65 mother–offspring pairs through genotype matching using the 12 best markers (including the 5 specifically designed for crested macaques).

### Investigating Paternity Distribution

#### Paternity Determination

We used the 65 mother–offspring pairs in paternity analysis. Our paternity dataset included all offspring born into the three groups between 2006 and 2011 that we could sample. Following a conservative approach, we assigned paternity only when exclusion and likelihood calculations revealed the same father (cf. Widdig *et al.* 2017). In our exclusion method, we assigned paternity to the male that had no mismatches with a given mother–offspring pair across all loci while all other potential sires mismatched the offspring at two or more loci (strict exclusion). We also assigned paternity to the male with no mismatches with a given mother–offspring pair across all loci while one or more males mismatched the offspring at one locus only (relaxed exclusion). We used the program FINDSIRE (https://www.uni-kiel.de/medinfo/ mitarbeiter/krawczak/download/) to establish paternity exclusion. We used the same set of males, i.e., all potential sires, to calculate likelihood-odds (LOD) scores and confidence levels and confirm sires using likelihood analyses in CERVUS 3.0. We used the following parameters in CERVUS: simulated offspring: 100; number of candidate fathers: 56; proportion of candidate fathers sampled: 0.96; proportion of loci typed: 0.99; proportion of loci mistyped: 0.01; minimum number of typed loci: 10. To assess the proportion of extragroup paternities, we checked whether the assigned sire was a member of the infant’s birth group at the time of the infant’s conception using demographic and hormonal data (A. Engelhardt, *unpubl. Data*). Given the delay in natal dispersal, we also investigated whether the assigned sire was natal to the birth group of the infant to detect cases of natal breeding using demographic data (A. Engelhardt, *unpubl. Data*).

#### Degree of Male Reproductive Skew

We determined the degree of male reproductive skew using Nonacs’ *B* Index (Nonacs 2000, 2003) with Skew Calculator 2003 (http://www.eeb.ucla.edu/Faculty/Nonacs/PI.htm). Positive values of the *B* index suggest that the skew is higher than expected, while negative values suggest that reproduction is more equally distributed than expected (Kutsukake and Nunn 2006). Furthermore, an index close to 0 indicates a random distribution of paternities across potential sires, whereas values close to 1 suggest a high monopolization of reproduction by a single male. The advantage of the *B* index is that it can incorporate the total number of days adult males spent in a given group per year. We included information on group membership in the skew calculation based on demographic data. The program also computes 95% confidence intervals (CIs), with the width of the confidence interval revealing the precision of the estimates. If the CI includes 0, then the distribution of paternity among group males is not significantly different from random.

As our sampling effort was not consistent across the study period, the skew analysis includes only years and groups in which we sampled at least 45% of offspring born (mean ± SD = 66.8% + 28.6%). Therefore, we restricted the skew analysis to offspring born between 2007 and 2009 in R1 and R2 and born in 2009 in PB, giving 51 offspring with solved paternity. Although crested macaques are only moderately seasonal, we calculated the annual skew per group and year. Ideally, we should determine the degree of skew in successful conceptions during each alpha tenure; however, the number of offspring conceived per alpha tenure was low owing to the typically short tenure (mean 12 mo; see Marty *et al.* 2015).

### Assessing Genetic Variation and Inbreeding

For each of the selected markers, we computed standard population genetic parameters of genetic variation within a population. First, we calculated the expected heterozygosity (He), defined as the probability that an individual in a population is heterozygous at a given locus. Second, we determined the observed heterozygosity (Ho) by counting the frequency of heterozygous individuals per locus. If the observed heterozygosity is lower than expected, this indicates inbreeding, while a higher than expected heterozygosity suggests a mixture of two previously isolated populations (Hartl and Clark 1997). Furthermore, we determined inbreeding coefficients (*F*_IS_), where positive values indicate a deficit of heterozygosity (i.e., inbreeding) while negative values indicate an excess of heterozygosity (Hedrick 2000). We conducted all calculations (including PIC and HWE) in CERVUS 3.0 (Kalinowski *et al.* 2007) except the Wright *F* statistics (*F*_IS_), which we computed in FSTAT (version 2.9.3.) (Goudet 2001).

#### Ethical Note

This research complied with protocols approved by the Indonesian Institute for Science and Technology (RISTEK) and the Indonesian Ministry of Forestry (PHKA) and adhered to the legal requirements of Indonesia and Germany. We received permits to collect samples and export DNA extracts from the Indonesian Ministry of Forestry. Furthermore, we carried out our research in compliance with the animal care regulations and the principles of the American Society of Primatologists and the German Primate Center for the ethical treatment of nonhuman primates. We collected fecal samples from wild and captive individuals noninvasively after the animals left the site without disturbing, threatening, or harming them in their natural behavior, and obtained blood samples as part of the regular health check.

The authors declare that they have no conflict of interest

## Results

### Identification of Polymorphic Markers

Overall, 31% (12/39) of the markers we tested were suitable for investigating the crested macaque population at Tangkoko. These included 10 tetranucleotide and 2 dinucleotide loci (Table I) with 4–9 alleles per locus (Table II). We typed 176 individuals at 12 ± 0.3 (mean ± SD) loci (Table II).

**Table II:** Number of alleles, observed and expected heterozygosity, polymorphic information content, and inbreeding coefficient for 12 selected markers overall (all) and per group (R1, R2, PB), with the mean and standard deviation (SD) across all markers

	Number of alleles				Observed heterozygosity				Expected heterozygosity				Polymorphic information content				Inbreeding coefficient			
Locus	All	R1	R2	PB	All	R1	R2	PB	All	R1	R2	PB	All	R1	R2	PB	All	R1	R2	PB
D1s548	6	5	6	5	0.784	0.726	0.833	0.881	0.736	0.726	0.765	0.736	0.697	0.681	0.725	0.690	−0.065	0.000	−0.090	−0.199
D3s1768	7	7	6	6	0.851	0.855	0.881	0.833	0.781	0.757	0.776	0.768	0.744	0.713	0.734	0.721	−0.089	−0.131	−0.137	−0.086
D5s1457	6	5	5	5	0.727	0.714	0.717	0.714	0.649	0.674	0.645	0.609	0.589	0.613	0.581	0.541	−0.121	−0.060	−0.112	−0.175
D6s493	5	4	5	3	0.688	0.683	0.627	0.780	0.643	0.648	0.614	0.658	0.579	0.580	0.553	0.577	−0.070	−0.054	−0.021	−0.190
D6s501	5	4	5	4	0.727	0.679	0.783	0.714	0.682	0.675	0.692	0.669	0.614	0.602	0.621	0.598	−0.067	−0.006	−0.133	−0.068
D7s2204	6	6	6	6	0.805	0.831	0.817	0.756	0.724	0.727	0.69	0.721	0.674	0.673	0.633	0.668	−0.112	−0.144	−0.185	−0.049
D10s1432	4	4	4	4	0.710	0.690	0.833	0.548	0.613	0.615	0.628	0.567	0.538	0.542	0.545	0.476	−0.159	−0.124	−0.332	0.035
D11s925	9	9	8	9	0.792	0.805	0.746	0.810	0.748	0.754	0.731	0.758	0.725	0.731	0.701	0.714	−0.059	−0.068	−0.020	−0.069
D12s67	9	9	8	7	0.856	0.869	0.879	0.762	0.818	0.825	0.779	0.806	0.790	0.796	0.735	0.768	−0.047	−0.054	−0.130	0.055
D13s765	7	7	7	6	0.795	0.762	0.800	0.810	0.727	0.691	0.703	0.762	0.693	0.655	0.656	0.713	−0.095	−0.104	−0.140	−0.063
D14s255	3	3	3	3	0.665	0.774	0.550	0.619	0.651	0.669	0.601	0.626	0.575	0.591	0.529	0.537	−0.021	−0.158	0.085	0.011
D18s536	6	6	5	5	0.787	0.771	0.767	0.805	0.723	0.711	0.705	0.702	0.672	0.655	0.651	0.635	−0.089	−0.085	−0.089	−0.149
Mean	6.1	5.8	5.7	5.3	0.766	0.763	0.769	0.753	0.708	0.706	0.694	0.699	0.658	0.653	0.639	0.637	−0.082	−0.082	−0.109	−0.079
SD	1.7	1.9	1.4	1.6	0.059	0.064	0.095	0.089	0.059	0.054	0.059	0.070	0.075	0.069	0.072	0.087	0.035	0.049	0.097	0.082

The analysis is based on 176 crested macaques from three groups in the Tangkoko population in North Sulawesi, Indonesia.

### Testing the Suitability of Selected Markers

#### Polymorphic Information Content and Hardy–Weinberg Equilibrium

The PIC ranged 0.538–0.790 with a mean of 0.658 ± 0.075 (mean ± SD) (Table II), suggesting our markers had high discriminating power. We detected no significant deviation from Hardy–Weinberg or evidence of null alleles (Table I).

#### Mendelian Inheritance

We confirmed all 65 maternities (assigned by behavioral observations) through genotype matching (65 pairs × 10–12 loci) with one mismatch in one mother–offspring pair.

### Investigating Paternity Distribution

#### Paternity Determination

Our dataset included 65 offspring for which we could solve 63 paternities (97%). In 40 cases, we excluded all males on at least 2 loci, except for the assigned sire, which matched the offspring–mother pair at all loci (strict exclusion). In 14 cases, the assigned sire had no mismatch with the respective mother–offspring pair, but we excluded the next candidate sire at only one locus (relaxed exclusion). In 8 further cases, the assigned sire had one mismatch with the given infant, while the next likely sires had at least two mismatches (best match). In one case, two males matched the infant–mother pair at all loci (tie) and both males were also present in the group around the conception of the infant. In this case, we accepted the male assigned by CERVUS (Kalinowski *et al.* 2007) as the sire. In all cases, CERVUS supported the sires assigned based on exclusion rules (95% confidence level; see Electronic Supplementary Material for an overview of genotypes and trios). In the remaining two cases, we did not assign paternity because the exclusion and likelihood approach did not reveal the same father. We found no evidence of extragroup paternity or natal breeding in the solved paternity cases.

#### Degree of Male Reproductive Skew

Although 18 males sired the 63 infants investigated, the mean male reproductive skew per group and year as assessed by the *B* index was relatively high (mean ± SD: 0.330 ± 0.267, range: 0.021–0.672). The *B* index was significantly different from a random distribution across groups and years, e.g., very high for all years in group R2, except for two of three years in group R1 (Table III). A posteriori analysis showed that the sex ratio (m/f) was negatively related to the *B* index; a female-biased sex ratio significantly increased the *B* index (Spearman ρ = −0.857, *N* = 7, *P* = 0.014) (Table III). Finally, the mean proportion of alpha paternity was 65% per year with high variation across groups (29–100%).

**Table III:** Degree of male reproductive skew in three groups of crested macaques at Tangkoko Reserve, Indonesia, 2007–2009

Group and year	Number of potential group sires	Number of group sires	Number of adult females	Number of determined paternities	Proportion of alpha-male paternity (%)	Proportion of alpha-male tenure across the year (%)	Observed *B* index	*P* level	Lower confidence interval	Upper confidence interval
R1 2007	15	4	20	9	55.56	73.15	0.179	**0.001**	0.033	0.455
R1 2008	20	2	21	3	33.33	73.42	0.139	0.165	−0.303	0.562
R1 2009	21	5	25	7	28.57	97.26	0.021	0.250	−0.133	0.289
R2 2007	14	3	18	9	77.78	18.38	0.527	**0.000**	0.192	0.865
R2 2008	7	1	19	7	100.00	100.00	0.672	**0.000**	0.214	0.672
R2 2009	10	1	20	9	100.00	100.00	0.621	**0.000**	0.251	0.621
PB 2009	16	3	17	7	57.14	31.51	0.153	**0.016**	0.016	0.506
Mean	14.7	2.7	20.0	7.3	64.63	70.53	0.330			
SD	5.0	1.5	2.6	2.1	29.14	33.44	0.267			

We provide the number of potential group sires, number of group sires, number of adult females, number of determined paternities, proportion of alpha-male paternity, proportion of alpha-male tenure across the year, the observed *B* value, the lower and upper confidence interval (each 0.95%) together with the *P* value that the observed *B* value is due to chance (significant values in bold). The B index incorporates male residency in days per group and year. This analysis includes a total of 51 offspring.

### Assessing Genetic Variation and Inbreeding

The observed heterozygosity (Ho) ranged from 0.665 to 0.856, and expected heterozygosity (He) from 0.613 to 0.818 (Table II). The mean observed heterozygosity (mean ± SD = 0.766 ± 0.059) was greater than the mean expected heterozygosity (mean ± SD = 0.708 ± 0.059) (Table II), suggesting no risk of inbreeding at this point in time in our study groups (see Hartl and Clark 1997, for comparison). In other words, although we expected around 70% of individuals to be heterozygous at a given locus under random mating conditions, on average ca. 76% of individuals were heterozygous. Similarly, the mean *F*_IS_ across the three groups was −0.082 ± 0.035 (mean ± SD), with *F*_IS_ consistently <0 for all 12 polymorphic loci, indicating an excess of observed heterozygosity (see Hedrick 2000, for comparison). In other words, individuals were less related than expected under random mating. Finally, we found no major differences between groups in terms of number of alleles per locus and degree of heterozygosity (Table II), suggesting comparable estimates of genetic variability despite different group size, degree of skew, and duration of alpha tenure.

## Discussion

Our results show that the 12 selected microsatellite markers provide reliable information on individual genotypes in crested macaques and are useful for various applications in field studies on this species. Specifically, they provided high confidence in paternity assignment, a relatively high level of polymorphic information content and genetic variation (assessed by heterozygosity and inbreeding coefficients), and a high accuracy of allele characterization, i.e., low occurrence or absence of mutations. Furthermore, they comprise mainly tetranucleotide repeats, which are usually easier to analyze and thus enhance the reliability of genotyping. Altogether, the selected markers fulfill important genetic and technical criteria that are critical for the precision and efficacy of high-throughput genotyping (Butler *et al.* 2001).

We report highly polymorphic markers in Sulawesi macaques. Although we used primers formerly applied to other macaque species, several markers did not generate satisfying PCR products. We thus modified specific primers for crested macaques that produced much more reliable amplification results. However, given that Sulawesi macaques split from their common ancestor with southern pig-tailed macaques from Borneo (*Macaca nemestrina*) only in the early to middle Pleistocene (Evans *et al.* 1999; Fooden 1969), most, if not all, of the loci used in this study are likely informative in the other Sulawesi macaque species too. With the validated markers and improved primers, we thus provide an important tool for conservation management to assess gene flow, heterozygosity, and inbreeding depression of small and/or isolated populations across the whole island. Furthermore, with this set of markers, we will be able to conduct more detailed studies of population genetics, sexual selection, behaviour, and sociobiology, including parentage data. We encourage the application of the selected markers to other Sulawesi macaque species.

We assigned paternity to 97% of offspring sampled with 95% confidence, demonstrating the high analytical power of the marker set and its usefulness for studies of sexual selection and reproductive success. Although we cannot draw conclusions for the two offspring with unsolved paternity, all cases of solved paternity show no indication of extragroup paternity and natal breeding. This is interesting, given that male crested macaques do not disperse until they fully developed, and their competitive ability is sufficient for challenging alpha males in nonnatal groups (Marty *et al.* 2015). Furthermore, groups are large enough for unrelated potential mates to coexist in the natal group. It thus seems that male crested macaques need to migrate and successfully take over the alpha position to reproduce (Marty *et al.* 2016). It is also surprising that we found no extragroup paternity. Adjacent groups meet frequently and groups are too large and the vegetation is too dense for males to oversee the whole group. This suggests that females ready to conceive are either well mate-guarded during intergroup encounters, or refrain from mating with nongroup males. More detailed behavioral observations during intergroup encounters are needed to show which of these two explanations hold true for crested macaques.

As predicted from mating observations, we found a skew in male reproduction toward alpha males. The mean alpha paternity was 65% and ranged 29–100% across years and groups. Similarly, the degree of skew varied considerably across groups. Notably, our study on crested macaques found the highest *B* index reported so far for any primate (maximum: 0.672, mean: 0.330). In a study of free-ranging rhesus macaques, the skew in one large group varied 0.049–0.106 across six consecutive years (Widdig *et al.* 2004) and in one small group, the mean *B* index was 0.084 over two consecutive years (Dubuc *et al.* 2011). In wild Assamese macaques (*Macaca assamensis*), the mean *B* index was only 0.087 over 6 yr. in one group, with the alpha share of paternity limited to 29% (Sukmak *et al.* 2014).

Takeover rates had a negative effect on reproductive skew. The largest group, R1, generally had a lower skew and was subject to frequent alpha takeovers, i.e., the male hierarchy was dynamic, while group R2 showed skew values as high as 0.672, but had fewer takeovers, i.e., extended alpha tenure. These data are in line with results from species with extraordinary long alpha tenures, such as capuchin monkeys (*Cebus capucinus*), with an observed *B* index calculated across eight alpha tenure periods varying from −0.125 to 0.473 (mean: 0.274) (Muniz *et al.* 2010). Similarly, mountain gorillas (*Gorilla beringei beringei*) showed *B* indices between 0.337 and 0.432 in four groups containing multiple males of long tenure (Bradley *et al.* 2005). It is surprising, however, that the skew in R2 study group was higher than in the gorilla study, where a single male usually monopolizes all reproduction in his group. Skew calculations across these three studies are comparable, as they were calculated over the timeframe of alpha male tenure typical for each species. In other words, for crested macaques with their extraordinary short alpha tenure we computed annual skew per group, while in the two other species with long tenure, skew was computed over multiple years of alpha tenure per group. One potential reason for the comparatively large skew in crested macaques is that male crested macaques need to maximize their reproductive effort in a short timeframe. Hence, alpha tenure length might affect the interspecific variation in reproductive skew. However, our study might also provide a potential explanation for the intraspecific variation in skew. A more female-biased sex ratio significantly increased the *B* index, which suggests that when more females are available, there is more room for a few males to successfully monopolize receptive females, in contrast to when more male competitors are present. This supports the hypothesis that enhanced male monopolization, among other factors, results in a higher degree of reproductive skew (Gogarten and Koenig 2012; Ostner *et al.* 2008).

The high degree of male reproductive skew observed in our study animals did not translate into lower genetic variation in the population than we would expect under random mating. This is interesting given that only a few dominant males pass their genes into the next generation. Most likely, the high rates of alpha male takeover reported for this population counterbalance this effect. We need more detailed data on genetic variation in relation to tenure length to understand this process better.

Our study individuals reflect a geographically isolated population of a Critically Endangered species, but our analysis indicates no recent threat of considerable loss of heterozygosis and/or of inbreeding depression in the study population. Compared to studies of other macaque species, mainly using different markers, e.g., *Macaca mulatta* (Bercovitch and Nürnberg 1997), *M. sinica* (Keane *et al.* 1997), *M. sylvanus* (Kümmerli and Martin 2005), *M. fuscata* (Inoue and Takenaka 2008), and *M. assamensi* (Sukmak *et al.* 2014), our markers were highly polymorphic. Despite the small population size, it is possible that males migrate in and out of the Tangkoko population, contributing to the genetic variability observed.

In contrast to our results, we found no polymorphism in a set of mtDNA markers in another study using a subset of the individuals included here, i.e., 12 females and 4 nonnatal males from two groups (A. Engelhardt, *unpubl. Data*). This could indicate that the population of Tangkoko may already be inbred or stems from one single matriline. To determine the degree of inbreeding in crested macaques at Tangkoko more precisely, we will need extended studies over a broader range of groups. Furthermore, we need studies investigating the links between reproductive patterns, genetic variation, and population demography over time to expand our understanding of viability of threatened populations in the wild.

In conclusion, we provide genetic markers useful for studies on the conservation management and evolutionary biology of crested macaques, and likely of Sulawesi macaques in general. Parentage analysis of these species can contribute insights into the relationship among social style, reproductive patterns, and relatedness among macaque species (Schülke and Ostner 2008). The fact that the Tangkoko population of crested macaques is still genetically variable despite its small size, isolation, and the species’ reproductive patterns gives hope that other endangered primate species living in small, isolated populations may also retain a healthy gene pool, at least in the short term. However, while the population in Tangkoko does not seem to be suffering from genetic depletion, other isolated populations of crested macaques might. With the described markers at hand, we will now be able to assess and manage genetic variation across all populations of crested macaques scattered over North Sulawesi. Data are shown in Supplementary Materials.

## Electronic supplementary material

ESM 1 (XLSX 36 kb)
